# A Multichannel Deep Neural Network for Retina Vessel Segmentation *via* a Fusion Mechanism

**DOI:** 10.3389/fbioe.2021.697915

**Published:** 2021-08-19

**Authors:** Jiaqi Ding, Zehua Zhang, Jijun Tang, Fei Guo

**Affiliations:** ^1^ School of Computer Science and Technology, College of Intelligence and Computing, Tianjin University, Tianjin, China; ^2^ School of Computer Science and Engineering, Central South University, Changsha, China

**Keywords:** retina vessel segmentation, multi-objective optimization, multiple probability map fusion mechanism, skeleton extraction, multi-channel DCNN

## Abstract

Changes in fundus blood vessels reflect the occurrence of eye diseases, and from this, we can explore other physical diseases that cause fundus lesions, such as diabetes and hypertension complication. However, the existing computational methods lack high efficiency and precision segmentation for the vascular ends and thin retina vessels. It is important to construct a reliable and quantitative automatic diagnostic method for improving the diagnosis efficiency. In this study, we propose a multichannel deep neural network for retina vessel segmentation. First, we apply U-net on original and thin (or thick) vessels for multi-objective optimization for purposively training thick and thin vessels. Then, we design a specific fusion mechanism for combining three kinds of prediction probability maps into a final binary segmentation map. Experiments show that our method can effectively improve the segmentation performances of thin blood vessels and vascular ends. It outperforms many current excellent vessel segmentation methods on three public datasets. In particular, it is pretty impressive that we achieve the best F1-score of 0.8247 on the DRIVE dataset and 0.8239 on the STARE dataset. The findings of this study have the potential for the application in an automated retinal image analysis, and it may provide a new, general, and high-performance computing framework for image segmentation.

## 1 Introduction

The fundus photography can quickly and noninvasively obtain retinal images, which is usually used as an effective way for diagnosing fundus diseases. Furthermore, by observing retina blood vessels, medical scientists can assess symptoms of diseases, such as hypertension, diabetes, and neurodegenerative diseases. However, many studies based on retinal vascular changes still rely on a manual qualitative assessment, which prevents experts from grasping retinal diseases more accurately and efficiently. For example, narrowed retinal blood vessels is a typical early symptom of hypertension, but disease symptoms can only be assessed subjectively by ophthalmologists through fundus photography or angiography. These early symptoms are not only time-consuming but also hard to be spotted. Therefore, a reliable and quantitative automatic diagnostic method is urgently required to improve diagnosis efficiency, and some related research works have gradually risen in the recent years.

Retina vessel segmentation methods are generally divided into filter-based methods, machine learning algorithms, and deep learning methods. The filter-based technology (Annunziata et al., 2016) is almost consistent with image processing methods, using the filter window to process fundus images. Peter et al. (2012) used a wavelet transform to quickly detect blood vessels and calculated vascular profiles to determine blood vessel boundaries. Fraz et al. (2012) employed the Gabor filter and top-hat transformations of morphological operations for feature extraction and vessel segmentation. Nguyen et al. (2013) performed vessel segmentations by linear operators of different scales. Salazar-Gonzalez et al. (2014) used graph cut technology for vessel segmentation. In addition, machine learning (Roychowdhury et al., 2014) models usually extract feature vectors and then construct a classifier to label pixels. Orlando and Blaschko (2014) used a conditional random field (CRF) with a fully connected model to segment the fundus retina vessels. Gu and Cheng (2014) proposed an iterative two-step learning-based method to boost the segmentation performance by existing basic segmenters. Lupascu et al. (2010) constructed a 41-D vector for each pixel in the image to encode the alignment information, and then classified pixels using the AdaBoost classifier.

With the rapid development of deep learning in recent years, convolutional neural network (CNN) performs well on classification and regression tasks because it can hierarchically abstract representations using local operations. It is very suitable for computer vision–related applications. Especially, since the advent of U-net (Ronneberger et al., 2015) in 2015, it brought great progress to medical image segmentation tasks. It is an encoder–decoder structure, and skip connections inspired many subsequent studies. For example, M2UNet by Laibacher et al. (2018) and LadderNet by Zhuang (2018) obtained excellent results in the fundus retina vessel segmentation. They both are inspired by U-net. In addition, Melinscak et al. (2015) developed a 10-layer CNN for a binary classification based on the patch-wise method. Fu et al. (2016) constructed a deeply integrated network consisting of a convolutional neural network (CNN) and a conditional random field (CRF). In detail, multi-scale and multilevel CNNs were used to extract features, and a CRF was used to model the pixel interaction. In the recent years, many researchers made great progress. CS-Net (Mou et al., 2019) adds two attention mechanisms: spatial attention and channel attention, to the encoder and decoder to better capture the local and global features of images, thereby improving the segmentation results. DUnet (Jin et al., 2019) integrated the deformable convolution into U-net so that it can adaptively adjust the receptive field of the filter during the feature extraction process to extract features of different scales. Vessel-Net (Wu et al., 2019) embedded the inception-residual convolution block into U-net to improve the feature extraction ability of the encoder, and then used multiple supervision paths to train the network to obtain more refined segmentation. Wang et al. (2020) separately trained the “easy” and “hard” parts in the encoder stage to perform targeted vascular segmentation, and added an attention mechanism to the “hard” part for more effective segmentation. NFN+ (Wu et al., 2020) used two networks to achieve more refined segmentation. It exploited the front network to obtain a basic prediction probability map, and then used the followed network for post-processing. In addition, the author applied inter-network skip connections to unite the two networks to make better use of multi-scale features. SCS-Net (Wu et al., 2021) first used a scale-aware feature aggregation (SFA) module to extract multi-scale features, then employed the adaptive feature fusion (AFF) module to fuse different levels of features to obtain richer semantic information, and finally used the multilevel semantic supervision (MSS) module to obtain more refined segmentation results. RV-GAN (Kamran et al., 2021) used a generative network to perform blood vessel segmentation. It employed two generators and two multi-scale discriminators for microvessel segmentation. In addition, it replaced the original adversarial loss with a new weighted loss.

However, the abovementioned methods are more focused on obtaining accurate prediction probability maps rather than binary segmentation features. But only increasing the accuracy of probability maps is very limited for the ability to improve the accuracy of segmentation. In addition, existing methods do not predict thick and thin vessels separately although they have different characteristics, which also leads to the relative neglect of improving accuracy on thin blood vessel segmentation. Therefore, we propose a specific method to skillfully fuse prediction results from original, thick, and thin vessels.

**FIGURE 1:**
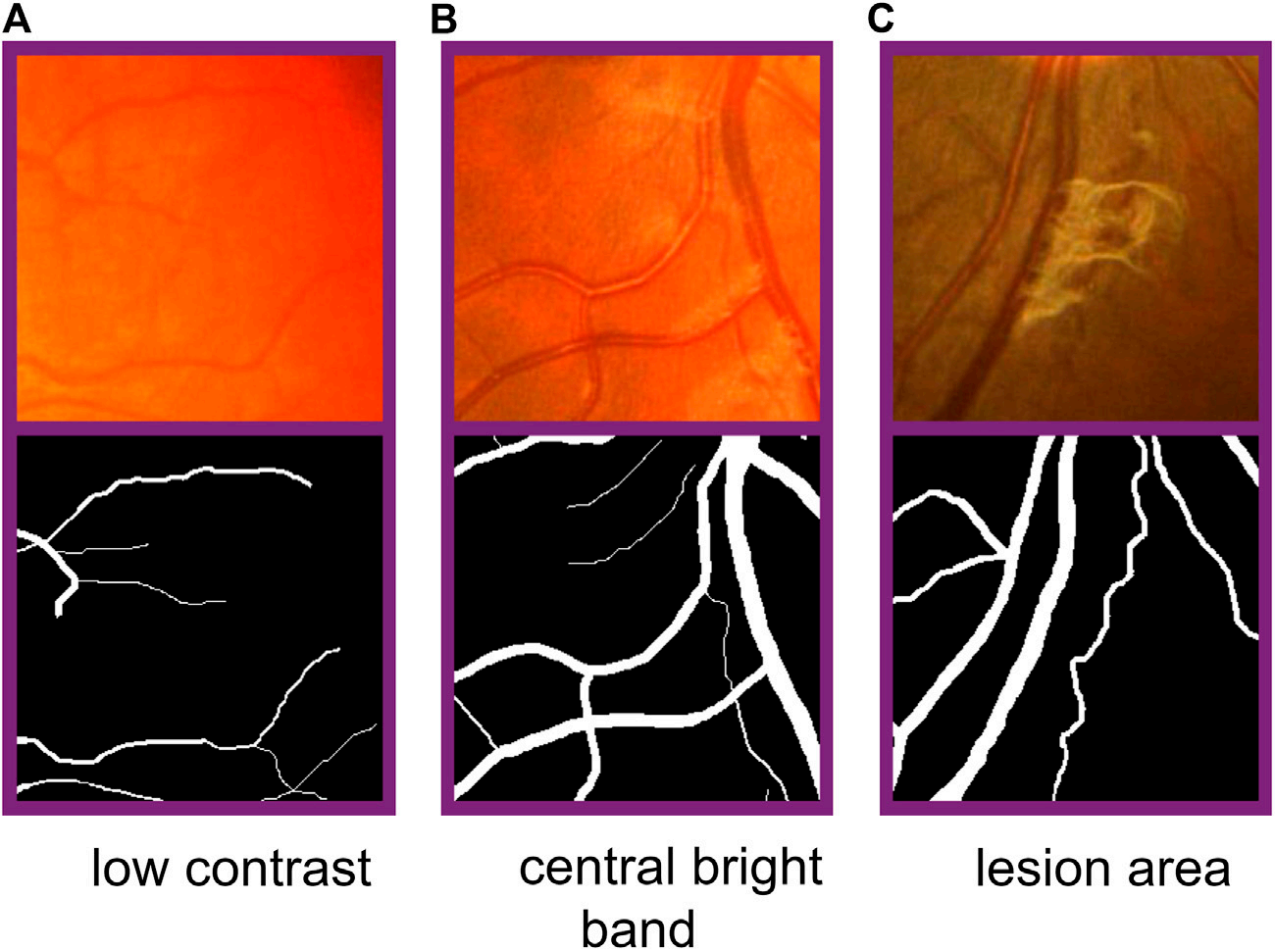
Difficulties in fundus image segmentation task. From the left to right: low contrast between blood vessels and background, central bright band in blood vessels, and lesion area around vessels. From the top to bottom: part of original images and corresponding labels.

In the task of retina vessel segmentation, there are many difficulties such as a low contrast between blood vessels and background, and central bright band in vessels and the lesion area around blood vessels, as shown in Figure 1. But segmenting vascular ends and thin blood vessels is the most difficult part. As we all know, the proportion of thin blood vessels in a retina image is small. So in the deep learning method, the misclassification or omission of some thin vessel pixels does not greatly affect the segmentation accuracy, but it leads to unsatisfactory segmentation maps, which causes the network to pay more attention to segment thick vessels than thin vessels. Inspired by this limitation, according to the original label and the other two extra training objectives made by ourselves, we train original, thick, and thin vessels separately. Thus, we can obtain three different prediction probability maps. Then we use a special fusion method instead of directly choosing a fixed threshold to get the final binary segmentation map. Three kinds of prediction probability maps can exert their own strength so that they can complement each other. Experimental results show that our training strategy and fusion mechanism can get excellent performance. Furthermore, our method can transfer our novel training strategy and fusion mechanism to other deep learning models, and they can perform better than before. Therefore, our proposed method can be commonly applied on any kind of deep learning models for retina image segmentation.

## 2 Related Works

With the development of computational technologies, various deep learning models have emerged for solving image calculation problems. AlexNet (Krizhevsky et al., 2012), which won the 2012 ImageNet competition, should be regarded as the first deep learning convolution neural network. It can extract higher dimensional features of images than LeNet (Lecun et al., 1998). The network structure was composed of eight layers, including five convolution layers and three fully connected layers. It also introduced the popular activation function ReLU (Rectified Linear Unit) and the Dropout layer used to prevent over-fitting. Also, VGGNet (Simonyan and Zisserman, 2014) is a very famous deep convolution neural network, which won the runner-up of the 2014 ILSVRC competition. It explored the relationship between the network depth and performance of the convolution neural network. What is more, ResNet (He et al., 2016) that won several championships in ILSVRC 2015 and COCO 2015 was dedicated to solve the model degradation problem caused by the deepening of the network during the layer stacking process. It used a shortcut connection to add the output of previous layers to the current output of this layer, and then the sum can be put into the activation function as the final output of this layer. It was proven that ResNet can effectively alleviate the problem of vanishing gradients.

In the field of image segmentation, there are still some methods that perform very well. The first to mention is fully convolutional networks (FCNs) (Shelhamer et al., 2017), which is a landmark invention in the field of image segmentation. It creatively replaced fully connected layers of the CNN with convolution layers. The FCN classified images at the pixel level, accepted images of any size, and obtained the output with the same size; thereby, it can solve the problem of image segmentation at the semantic level. There was also another characteristic, the skip-level structure, which can take into account local and global information simultaneously. Next one is SegNet (Badrinarayanan et al., 2017), a deep network to solve the problem of image semantic segmentation for autonomous driving or intelligent robots. Based on the semantic segmentation model of the FCN, the framework of VGG16 was used, and it removed the fully connected layer to build an encoder–decoder symmetrical structure to achieve end-to-end pixel-level image segmentation. One of its highlights was the use of max-pooling indexes, which can reduce the amount of parameters for end-to-end training and can be incorporated into any encoding–decoding architecture with only a few modifications. Finally, there is Mask R-CNN (He et al., 2017) that won the championship of COCO 2016 competition; it can perform instance segmentation while performing target detection. Mask R-CNN was based on Faster R-CNN (Ren et al., 2017). In Mask R-CNN, the FCN was used in semantic segmentation for each proposal box of Faster R-CNN. In addition, another important change was the replacement of the ROI pooling module of Faster R-CNN with a more accurate ROI align module.

## 3 Methodology

**FIGURE 2:**
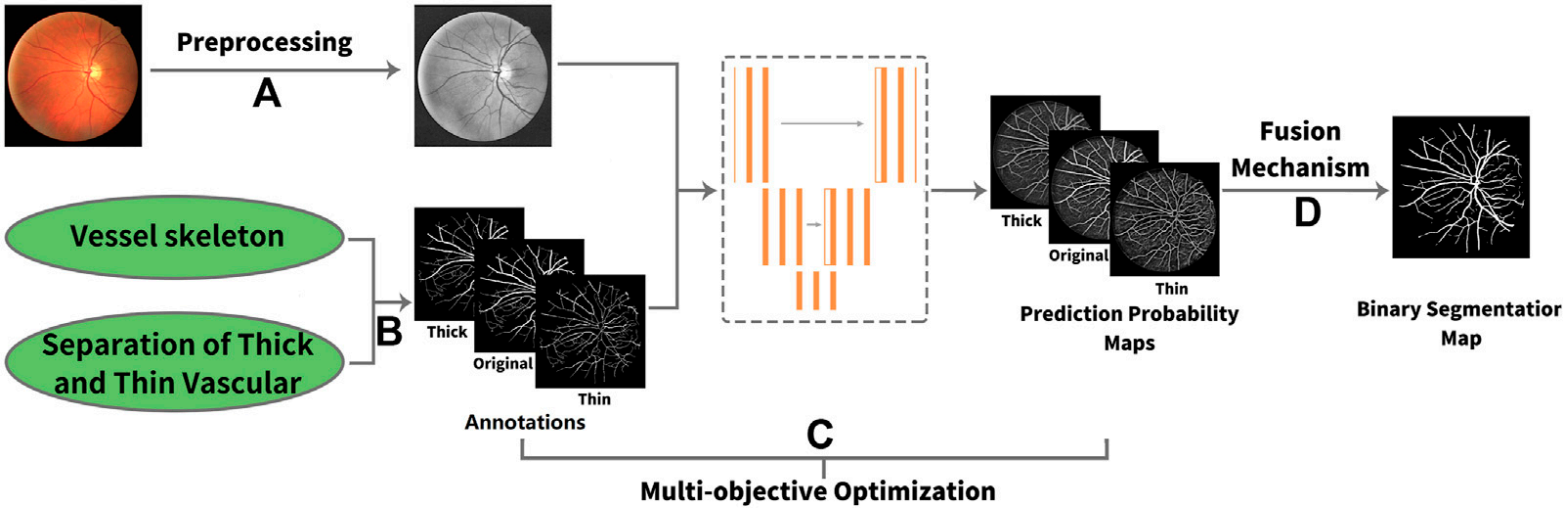
Framework of our method. **(A)** Process of preprocessing. **(B)** Original label, thick, and thin training objectives. **(C)** Multi-objective optimization. Three types of annotations lead to three different prediction probability maps. **(D)** Fusion mechanism.

In this article, we propose a novel deep learning framework for fundus diseases diagnosis. First, the fundus images were preprocessed and divided into patches. Second, we perform multi-objective optimization on the network. Explaining in detail, given an image, there is the original label, and then the extra thick and thin vascular training objectives can be obtained by our own algorithms. Based on three different annotation sets, we can obtain the prediction probability maps of original, thick, and thin vessels. Finally, our new fusion mechanism fuses three different prediction probability maps to obtain the final binary segmentation map. The framework of our method is shown in Figure 2.

### 3.1 Preprocessing

**FIGURE 3:**
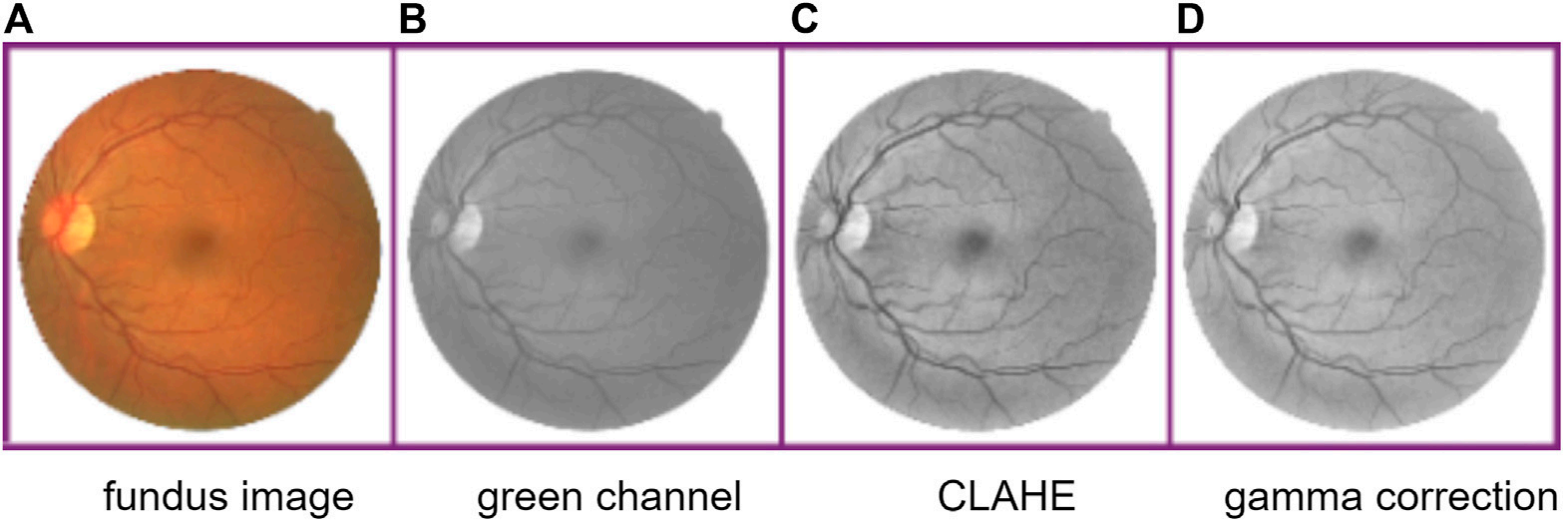
Process of image preprocessing. From the left to right: fundus retina image, image of green channel, CLAHE-processed image, and gamma correction processed image.

The fundus retina blood vessel images have uneven brightness, image noises, and low contrast between vessels and background. Thus, similar to other methods, we first extract the green channel of original RGB images because the green channel has the highest image contrast. Second, different images are normalized. Third, we apply contrast limited adaptive histogram equalization (CLAHE) (Zuiderveld, 1994) on these images so that the background brightness of images can be equalized without magnifying the image noise. Finally, the gamma correction is used to compress highlight portion and expand dark portion of images. The process of preprocessing is shown in Figure 3.

### 3.2 Network Architecture

**FIGURE 4:**
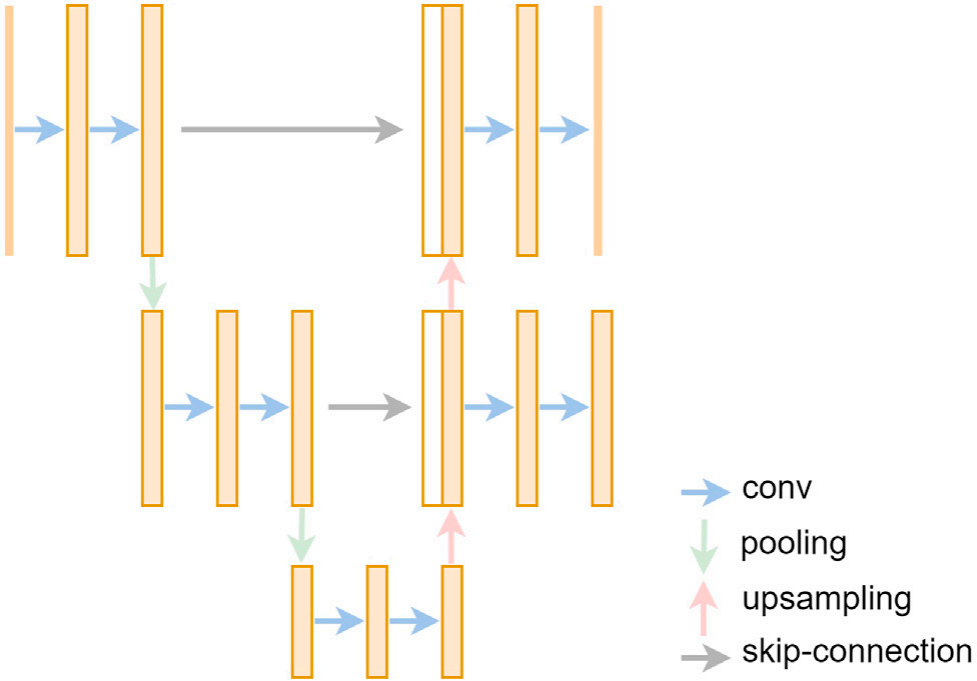
The structure of U-net.

U-net Ronneberger et al. (2015) has outstanding performances in medical image segmentation tasks. It was proposed at a medical image conference (MICCAI) in 2015. As shown in Figure 4, U-net had a symmetrical encoder–decoder structure. It performed feature extraction in the encoding stage. It uses convolution to gradually extract features of different depths. The convolution operation can be formulated as follows:

ConVk=ReLU{∑n=0N∑n=1NW(k)n,n∗X}. (1)

Here, *N* is the size of weight matrix *W*, *X* is the tensor from last layer, and ReLU is the rectified linear function; its expression is as follows:

$$ReLU=\{\begin{array}{cc} x & \text{x}\geq 0\\ 0 & \text{x}<0 \end{array},$$ (2)

Then the pixel-level classification can be obtained in the decoding stage *via* up-sampling operation. U-net also used skip-connection to concatenate features of the corresponding layers of the encoder and decoder on the channel dimension (see the gray arrows in Figure 4) so that deep semantic information and shallow representation information can be combined to make the segmentation results more refined. U-net performs very well in segmentation tasks of various organs, especially for fundus blood vessels; it can segment almost all thick blood vessels and most of thin blood vessels, achieving high accuracy. So we choose a U-net–based network as our segmentation model.

### 3.3 Multi-Objective Optimization

We construct a train model for original vessel images using preprocessed raw images, original vessel labels, and masks. Then, prediction probability maps of original vessels can be identified. Similarly, in order to achieve the specialized training on thin vessels and thick vessels separately, we use thin (or thick) vessel training objectives made by ourselves, which can emphasize thin (or thick) vessels relatively during the training process. Thus, thin (or thick) vessel training objectives, preprocessed images, and masks can be used as an input, and we can obtain prediction probability maps of thin (or thick) vessels.

**FIGURE 5:**
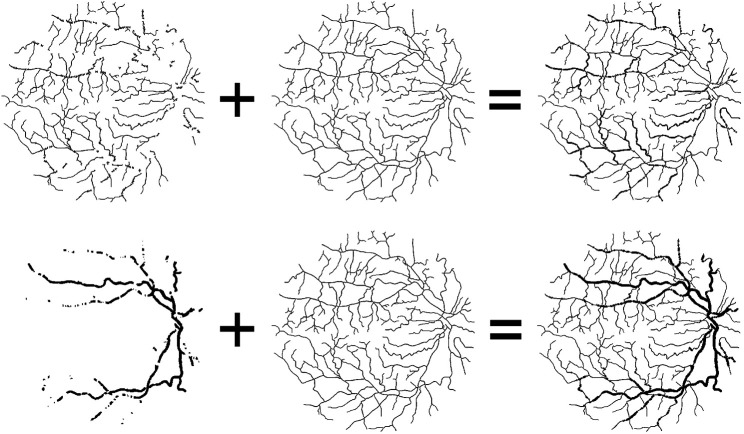
Composition of thin and thick vascular training objectives. From the left to right: thin (or thick) vessel of original label image, the same vessel skeleton, and thin (or thick) training objectives.

Here, the thin (or thick) vessel training objectives are composed of two parts: thin (or thick) blood vessels and vessel skeleton, as shown in Figure 5. For the thick vessel training objective, we retain the thick vessels of the original label image, remove the thin blood vessels, and replace it with the vessel skeleton. In the same way, the thin vessel training objectives are composed of the thin vessels and vessel skeleton. The reason why we use skeleton to replace the removed vessels is to preserve the complete vessel topology and keep vessels consistent during the training process.

#### 3.3.1 Vessel Skeleton

There are many existing methods to obtain the skeleton of an object in an image (Zhang and Suen, 1984; Saeed et al., 2010). Due to the uneven thickness and tortuosity of retina blood vessels, some previous methods are not very suitable for obtaining the skeleton of retina vessels. However, the vessel skeleton is needed as a vascular topology consisting of the centerline of blood vessels.

**FIGURE 6:**
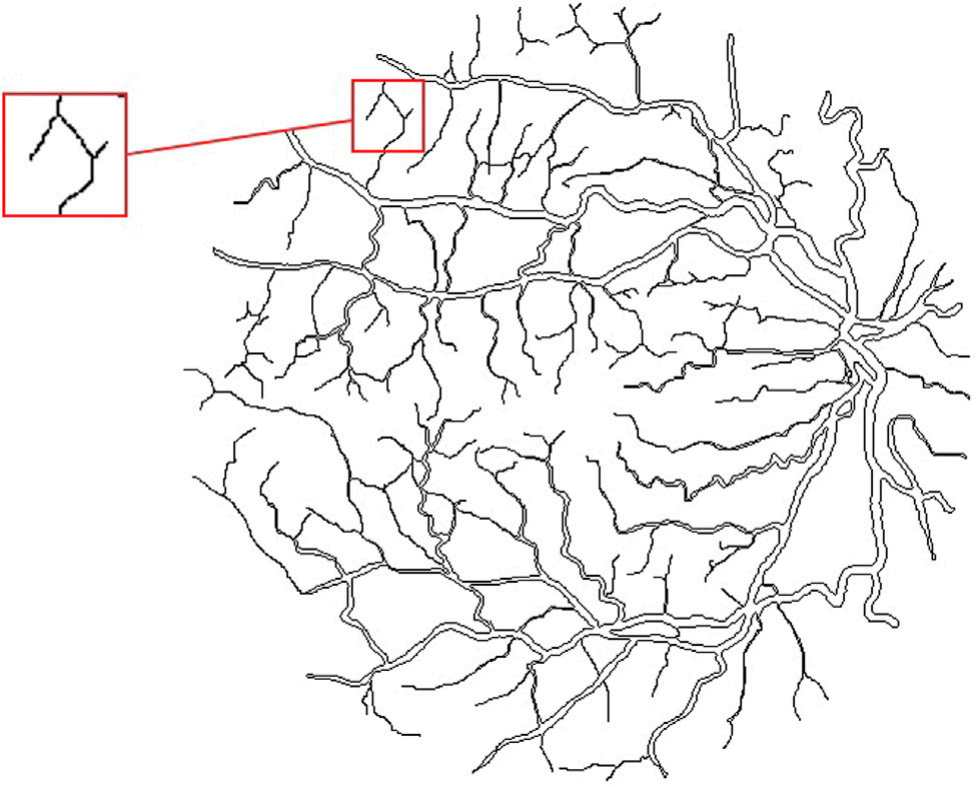
Outline of vessel. An exemplar of non-deletable points is shown in the red box: the vessel in this segment is only about two pixels wide. If removing the outline of this segment, the corresponding vessel in this segment will disappear.

**FIGURE 7:**
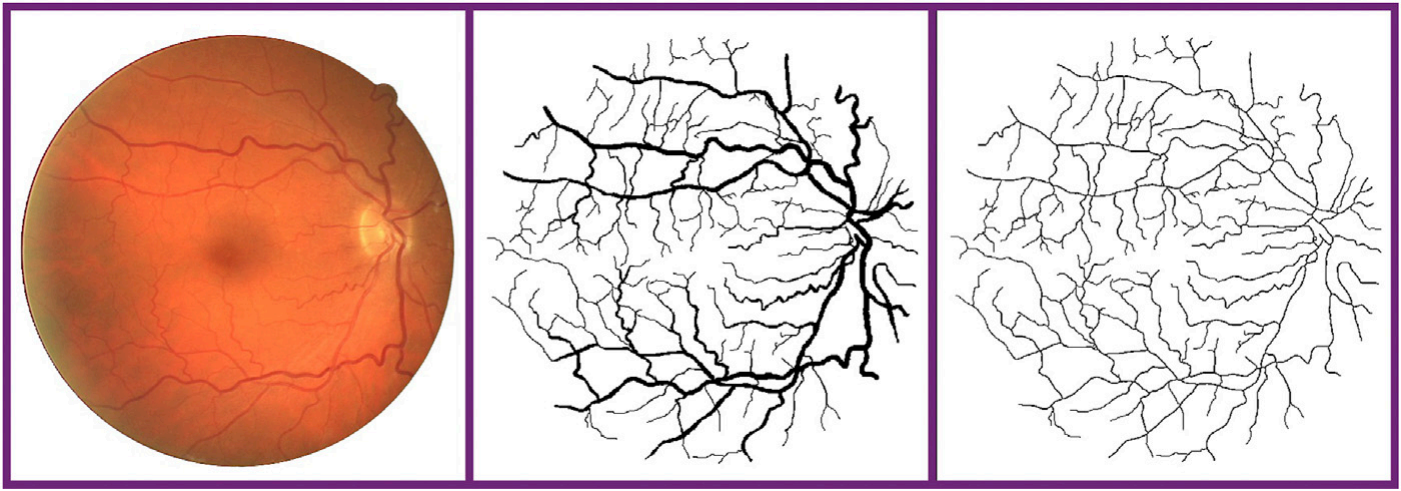
Vessel skeleton. From the left to right: fundus retina image, ground truth, and vessel skeleton obtained by our method.

Here, we get the retina vessel skeleton as follows. First, finding the outline of blood vessels. Through searching points with a pixel value of zero around each pixel of blood vessels, the boundary pixels of vessels can be identified. From this, we can define the outline of vascular. In other words, the outline is made up of all the boundary pixels, as shown in Figure 6. Next, except for the end pixels of blood vessels, rest of outline pixels are removed, having a new outline according to the above method. Our skeleton method keeps iterating this process until the vessel skeleton is obtained, that is, the horizontal width of the remaining blood vessels is less than two pixels. The vessel skeleton is shown in Figure 7.

#### 3.3.2 Separation of Thick and Thin Vessels

Because blood vessels gradually taper from the root to ends, the width of the vessel is gradually reduced. For each pixel in blood vessels, we match a vascular width to it, and then thick and thin vessels can be divided on the pixel level by the vascular width of every pixel. If the vascular width of any pixel is greater than or equal to the threshold, we define it as a thick vessel pixel, and naturally the opposite as thin vessel pixels. In detail, the vascular width at a certain point is defined as follows. First, we identify the vascular width of skeleton pixels. We define the twice distance from each skeleton pixel to the nearest outline pixel as the vascular width of this skeleton pixel. Second, we define the vascular width for other vascular pixels. The vascular width of the nearest skeleton pixel from them is used to replace their width. In this way, we can match the vascular width for each pixel. Thereby, thick and thin blood vessels are separated based on the vascular width of each pixel. In our experiments, the separation threshold is 2.2 pixels. This means that those with a width greater than or equal to 2.2 pixels are thick vessel pixels, and those with width less than 2.2 pixels are thin vessel pixels. Due to the slight uneven thickness of the blood vessels, this may lead to the intersection of separated thick and thin vascular pixels and unsmooth appearance of separation profile.

### 3.4 Fusion Mechanism

We design three fusion methods for prediction probability maps of original, thick, and thin vessels, and then adopt one of them that performs the best. For this method, we apply the pixel-wise classification on prediction probability maps. At a certain pixel point, if the pixel probability of one of three probability maps is greater than or equal to the threshold 0.5, it is defined as a vascular pixel. Furthermore, since the original vascular label is more complete, it has more complete segmentation map, accordingly. So during the fusion process, there is a greater weight on prediction probability maps of original vessels, and the best weight we get on the data set is 1.25; that is, *k* is 1.25. Therefore, we are relatively more strict with the application of prediction probability maps of thick and thin vessels, as shown in Algorithm 1.

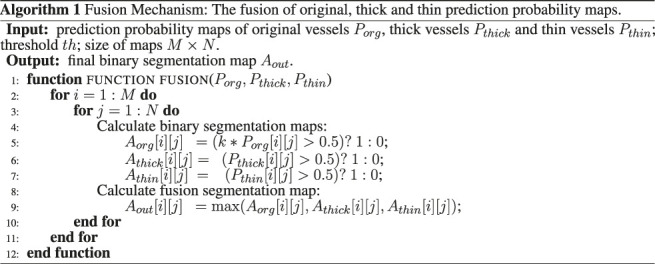

## 4 Experiments

In order to verify the validity of our method, we perform experiments on three datasets: DRIVE, STARE, and IOSTAR. Our experiments are implemented on Keras based on TensorFlow with GeForce RTX2080 Ti GPU. The network models for three tasks use the same parameter settings.

### 4.1 DRIVE Dataset

The DRIVE dataset contains 40 fundus images, corresponding labels, and binary field of view (FOV) masks. The images have the resolution of 584×565 pixels and $45^{\circ }$ FOV. We use the standard split: first 20 images belong to the test set and rest 20 images belong to the training set. Each image of the training set has one manual annotation, and each image of the test set has two manual annotations. In our experiments, the first manual annotation is used to be the gold standard.

### 4.2 STARE Dataset

The STARE dataset contains 20 fundus images and corresponding labels (Hoover et al., 2000). The images have the resolution of 605×700 pixels, 8 bits per color channel. This dataset does not contain FOV masks, so we use the masks provided by Marin et al. (2011) for comparison. Due to the small size of the dataset, we use leave-one-out method: select 19 images for training at a time, leave one image for testing, and then calculate the average of various metrics on 20 images as the final results. Similarly to other methods, we use the manual annotation by the first observer as our ground truth.

### 4.3 IOSTAR Dataset

The IOSTAR dataset contains 30 fundus images, corresponding labels, binary field of view (FOV) masks, and the optic disc (OD) masks. The images have the resolution of 1024×1024 pixels and $45^{\circ }$ FOV. Since the annotations of vessels within the OD are not available as stated on the dataset website, we take the official recommendation and use the OD mask for the evaluation of the retinal vessel segmentation. To make it easy for comparison, we also use five-fold cross-validation to train and test our model; that is, we select 24 images as the training set each time, then use the remaining six images to test, and finally use the average of the five tests as the final result.

## 5 Results and Discussion

In order to evaluate the segmentation method proposed in this article, we test on three datasets: DRIVE, STARE, and IOSTAR. First, so as to prove the model independence of our method, U-net is replaced with the FCN and the relevant experiments performed. Second, we compare the performances of four loss functions and list the relevant comparison results. Third, the other two fusion methods are introduced, and we compare them to our adopted fusion method. Then to verify the robustness of our method, we perform cross-training on two datasets: DRIVE and STARE. Finally, we compare our segmentation results with some existing methods.

### 5.1 Evaluation Metrics

Through the comparison between segmentation map and ground truth, the pixels which are in the segmentation map can be divided into the following four categories: correctly classified as positive (TP), correctly classified as negative (TN), incorrectly classified as positive (FP), and incorrectly classified as negative (FN). We use some general evaluation metrics such as Acc (accuracy), Se (sensitivity), Sp (specificity), and F1 score, as follows:

$$Acc=\frac{TP+TN}{TN+TP+FN+FP},$$ (3)

$$Se=\frac{TP}{TP+FN},$$ (4)

$$Sp=\frac{TN}{TN+FP},$$ (5)

$$F1=2\times \frac{TP}{TP+FP+FN}.$$ (6)

To further evaluate the effectiveness of our method, we also calculate the area under the receiver operating characteristics curve (AUC).

### 5.2 Performance on Fully Convolutional Networks

**FIGURE 8:**
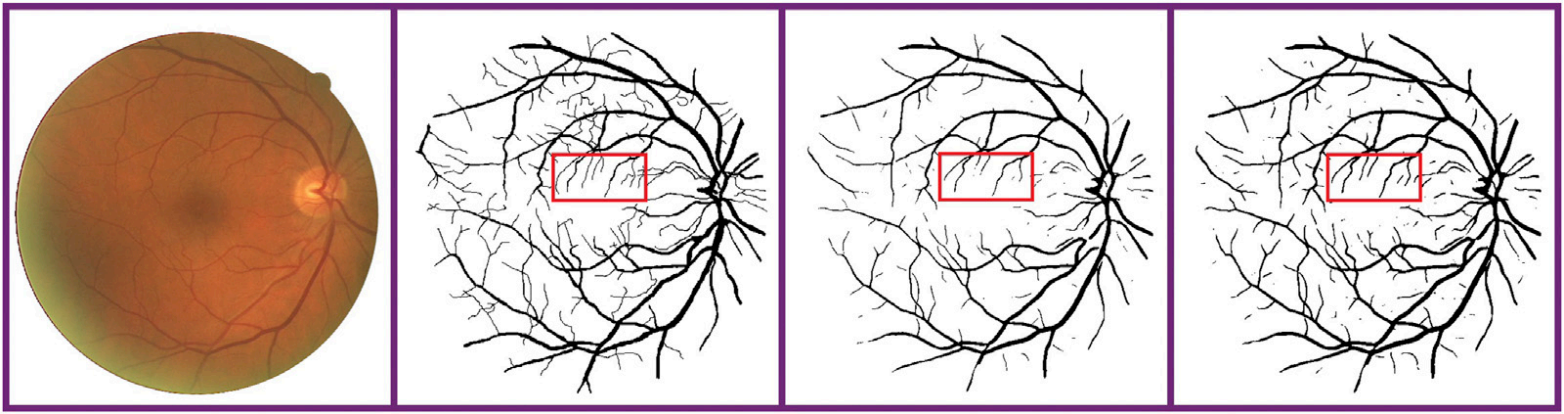
Comparison of a normal FCN-based model and our proposed method on the FCN-based model. From the left to right: fundus retina image, ground truth, binary segmentation map of the normal FCN-based model and our proposed method on the FCN-based model. Obviously, our method can segment more thin blood vessels.

To verify the model independence of our method, we replace U-net with the FCN model; that is to say, we transfer our training strategy and fusion mechanism to the FCN: first, training the original vessel images on the FCN-based model, then just as common methods, taking 0.5 as the threshold of the prediction probability map to turn it into a binary segmentation map. For comparison, we train original, thick, and thin vessels separately on the FCN-based model, and then apply our proposed fusion method to get a final segmentation map. We perform this comparison experiment on the DRIVE dataset, and related experimental results are shown in Figure 8. It can be found that our novel method can segment more vessel ends than the original normal FCN model. We achieve 0.8082, 0.9830, 0.8141, and 0.9677 on Se, Sp, F1-score, and Acc, respectively, while the normal FCN achieves 0.7718, 0.9795, 0.8072, and 0.96535 on Se, Sp, F1-score, and Acc, respectively.

### 5.3 Comparison of Loss Functions

We compare four loss functions on the basis of our framework, namely, binary cross entropy, categorical cross entropy, binary focal loss, and categorical focal loss. In deep learning methods for retinal vessel segmentation, the cross-entropy loss function is generally used. For the binary classification problem such as blood vessel segmentation, the first choice we think of is binary cross entropy. This loss can be defined as follows:

$$L_{bce}=\{\begin{array}{cc} -\log y^{\prime }, & \text{y}=1\\ -\text{log}\left(1-y^{\prime }\right), & \text{y}=0 \end{array}.$$ (7)

Here, *y* is the ground truth and $y^{\prime }$ is the prediction.

At the same time, for such problems, we can also use categorical cross entropy loss; the expression is shown as follows:

$$L_{ce}=-y*\text{log}y^{\prime },$$ (8)

where *y* is the ground truth and $y^{\prime }$ is the prediction.

The performances of above two cross-entropy loss functions do not have absolute strengths or weaknesses. According to the expression, it can be found that for all pixels in an image, whether they belong to foreground or background, the cross-entropy loss treats them all the same. Therefore, in the retina vessel segmentation tasks, even if thin vascular pixels with the small portion are not well segmented, cross-entropy loss will not be very high. So, it would place the emphasis on thick vessels and ignore thin vessels relatively.

In order to solve above problems, the focal loss (Lin et al., 2017) is a more appropriate choice. It can improve the accuracy of difficult segmented pixels. Focal loss can adjust a loss through two parameters, α and γ. α is the weighting factor; it can control the contribution of positive and negative samples to the total loss. And γ is the focusing parameter; its purpose is to reduce the weight of samples that are easy to classify so that the model can focus more on samples that are difficult to be classified during the training process. Inspired by the cross-entropy loss, we also try two different focal losses: binary focal loss and categorical focal loss. The expressions are as follows:

$$L_{binary\_focal}=\{\begin{array}{cc} -\alpha *{\left(1-y^{\prime }\right)}^{\gamma }\text{*log}y^{\prime }, & \text{y}=1\\ -\left(1-\alpha \right)\text{*}y^{\prime \gamma }\text{*log}\left(1-y^{\prime }\right), & \text{y}=0 \end{array},$$ (9)

$$L_{categorical\_focal}=-\alpha *\text{log}y^{\prime }\text{*}{\left(1-y^{\prime }\right)}^{\gamma }.$$ (10)

Here, *y* is the ground truth and $y^{\prime }$ is the prediction.

Here, α can play the role of balancing class. In categorical focal loss, α is a 2-dimensional vector because our task is a binary classification. In the binary focal loss, α is a fixed value.

**FIGURE 9:**
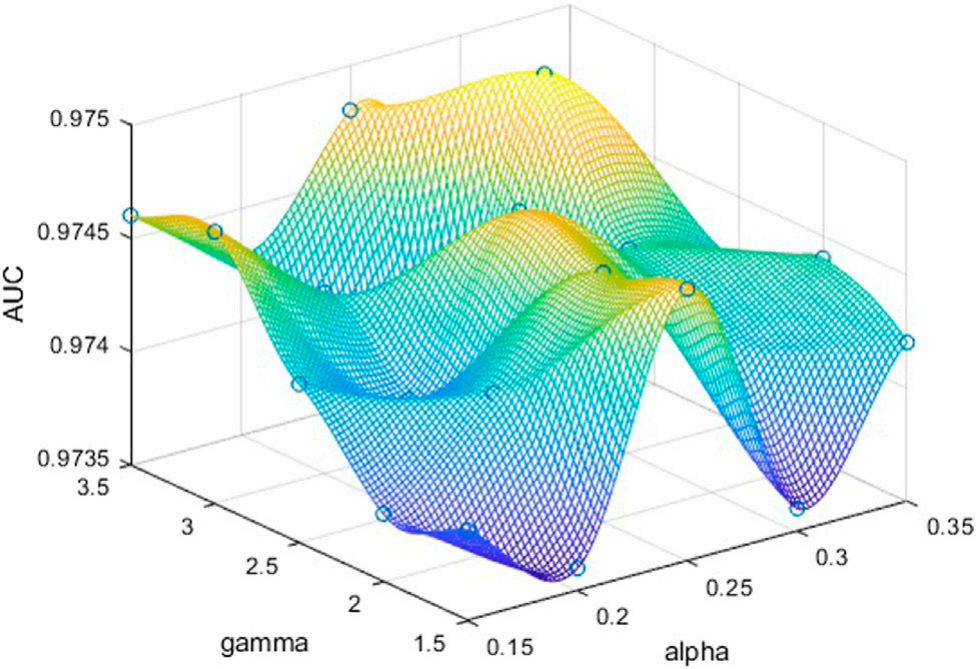
AUCs obtained by binary focal loss on DRIVE according to different α and γ. We can obtain the best performance when α is 0.3 and γ is 3.0.

**FIGURE 10:**
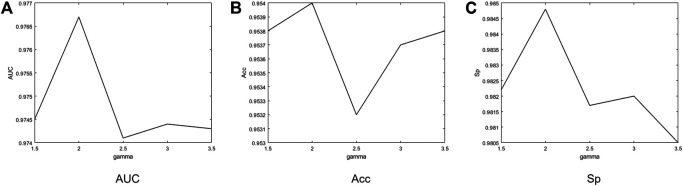
Performance obtained by categorical focal loss on DRIVE according to different γ. When γ is 2.0, the best values of AUC, Acc, and Sp can be obtained.

In order to further explore the role of α and γ in two focal loss functions, we take different values of α and γ to determine the best set of parameters. The experiments are performed on the DRIVE dataset. For binary focal loss, we compare α and γ, respectively. In the original article, the best α and γ are 0.25 and 2.5, respectively. Based on this, the range of α is 0.15−0.35, and the range of γ is 1.5−3.5, as shown in Figure 9. Since other metrics are related to the threshold of binary images, we chose to compare AUC that is just related to the predicted probability value. When *α* is 0.3 and γ is 3.0, the maximum AUC is 0.9750. Otherwise, there is no big difference between AUCs obtained by different parameters. For the categorical focal loss, we set α to 0.25 for positive samples directly and compare the performances of different γ. As shown in Figure 10, we can see that when γ is 2.0, the best values of AUC, Acc, and Sp can be obtained. So we can basically conclude that in our experiments, categorical cross entropy performs best when γ is 2.0.

**FIGURE 11:**
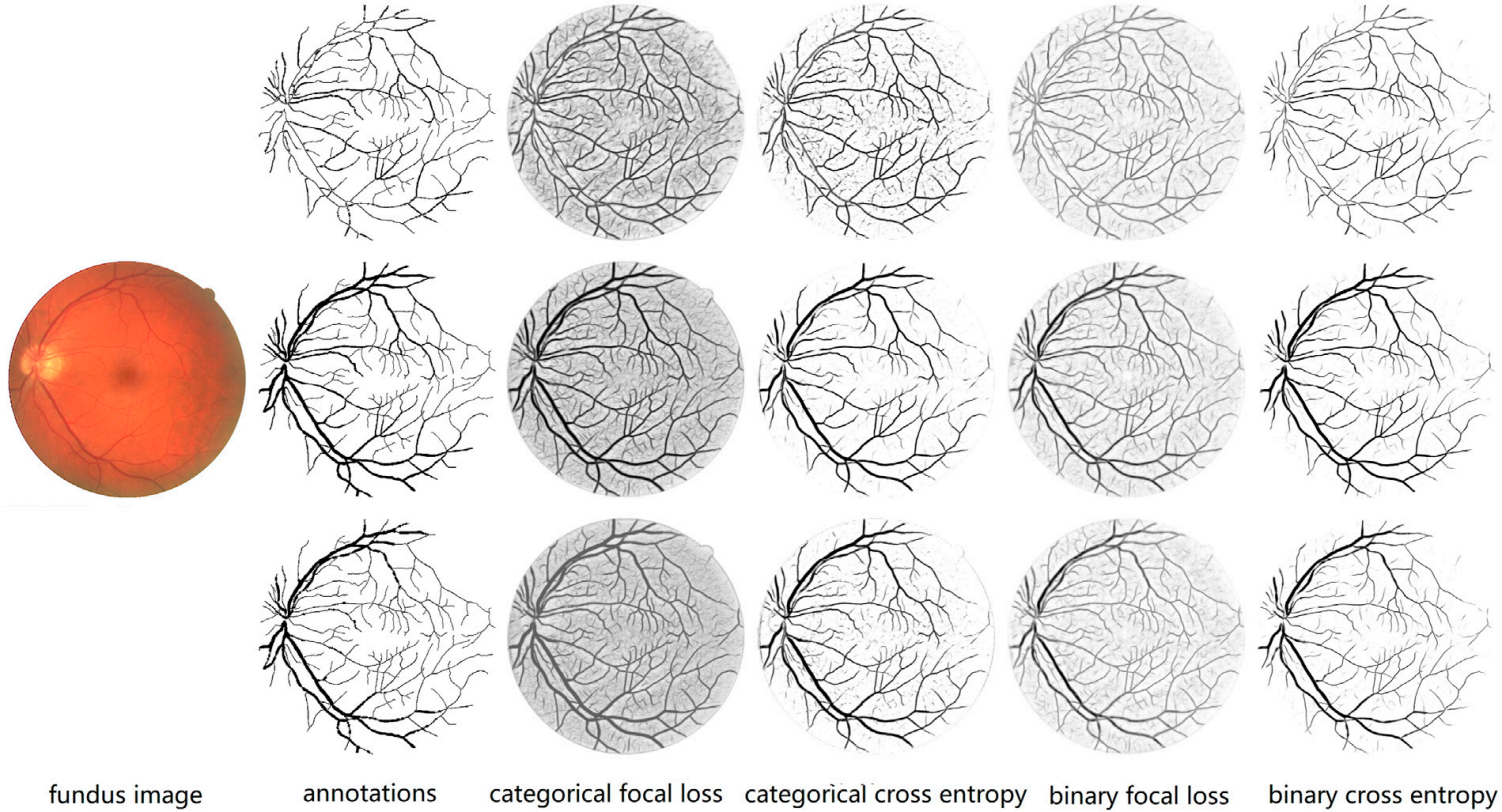
Comparison of different loss functions. From the left to right: fundus retina image, annotations of thin, original and thick vessels, prediction probability maps of thin, original, and thick vessels through categorical focal loss, categorical cross entropy, binary focal loss, and binary cross entropy, respectively. We can notice that categorical cross entropy is the best one for our proposed method because it can help us segment blood vessels more clearly, especially thin blood vessels.

We also compare the performances of four types of loss functions on original, thick, and thin blood vessels. For controlling variables, 0.5 is simply used as the threshold for segmentation instead of our proposed fusion method. For two types of focal loss, we use best parameters discussed above; that is, γ is 2.0 in categorical focal loss, and γ is 3.0 and α is 0.3 in the binary focal loss. As described in Table 1, for original blood vessels, except for categorical cross entropy with the best performance on Sp, the categorical focal loss realizes the best performances in the remaining metrics. For thick blood vessels, similarly, except for binary cross entropy with the best performance on Se, categorical focal loss achieves the best performances on Sp, F1-score, Acc, and AUC. However, for thin blood vessels, binary cross entropy performs the best on Se, F1-score, and AUC, while categorical focal loss achieves good results on Sp and Acc. In general, categorical cross entropy is the best one for our proposed method. Therefore, we choose categorical focal loss as our loss function. The performances of four loss functions are shown in Figure 11. At the same time, it can be noticed that when we train thick and thin vascular images, all results we get are lower than those of original vascular images. This is mainly because training objectives of thick and thin vessels do not exactly coincide with their original retina images.

**TABLE 1:** Performance on four loss functions.

Vessel Type	Loss	Se	Sp	F1	Acc	AUC
Original	Binary cross entropy	0.7586	0.9820	0.8061	0.9535	0.9750
	Categorical cross entropy	0.7074	**0.9867**	0.7866	0.9511	0.9735
	Binary focal	0.7637	0.9815	0.8079	0.9538	0.9750
	Categorical focal	**0.7677**	0.9816	**0.8107**	**0.9544**	**0.9782**
Thick	Binary cross entropy	**0.6917**	0.9791	0.7387	0.9493	0.9665
	Categorical cross entropy	0.6317	0.9825	0.7087	0.9462	0.9603
	Binary focal	0.6721	0.9814	0.7333	0.9494	0.9653
	Categorical focal	0.6846	**0.9877**	**0.7404**	**0.9559**	**0.9684**
Thin	Binary cross entropy	**0.6324**	0.9793	**0.6801**	0.9504	**0.9606**
	Categorical cross entropy	0.6091	0.9768	0.6536	0.9462	0.9506
	Binary focal	0.6233	0.9805	0.6785	0.9508	0.9589
	Categorical focal	0.6120	**0.9817**	0.6751	**0.9509**	0.9582

### 5.4 Comparison of Fusion Methods

We design two other fusion methods to compare with the first method mentioned earlier. In the other two methods, for a certain pixel in the image, its prediction values are first checked in the original, thick, and thin vascular prediction probability maps. Then for the second fusion method, if two of three prediction values are greater than or equal to the threshold 0.5, the pixel is determined as positive, that is, a blood vessel pixel, and its pixel value is set to 255 to obtain a binary segmentation map. And for the third method, we calculate the average of three prediction pixel values; if the average value is greater than or equal to the threshold 0.5, the pixel is defined as a vascular pixel. Comparison of three fusion methods is shown in Table 2. We can find that although the first fusion method is slightly lower than the third fusion method on Se, it performs best on Sp, F1-score, Acc, and AUC. So we finally chose the first fusion method as our fusion mechanism.

**TABLE 2:** Performance on the three fusion mechanisms.

Fusion	Se	Sp	F1	Acc
1st	0.8191	**0.9831**	**0.8201**	**0.9685**
2nd	0.8158	0.9820	0.8138	0.9673
3rd	**0.8201**	0.9821	0.8158	0.9677

### 5.5 Cross-Training on Different Datasets

To test the robustness of our method, we perform cross-training on two datasets: DRIVE and STARE. Similar to other methods (Yan et al., 2018), we first use the STARE dataset for training, and then test on the DRIVE dataset. In the same way, in turn, we use the DRIVE dataset for training and the STARE dataset for testing. The performances of cross-training are shown in Table 3. It can be found that for DRIVE (trained on STARE), our method obtained best results on Se, Sp, and Acc. And for STARE (trained on DRIVE), our method still obtained best results on Se, Sp, and Acc. However, we can also notice that results obtained by DRIVE (trained on STARE) are generally lower than those obtained by STARE (trained on DRIVE). Due to differences in manual annotations of two datasets, the model trained on STARE is relatively weak in segmenting images of DRIVE datasets because it is not able to detect thin vessels well.

**TABLE 3:** Cross-training results on DRIVE and STARE datasets.

Dataset	Method	Se	Sp	Acc	AUC
DRIVE (trained on STARE)	Marin et al.	—	—	0.9448	—
	Fraz et al.	0.7242	0.9792	0.9456	**0.9697**
	Li et al.	0.7273	0.9810	0.9486	0.9677
	Yan et al.	0.7292	0.9815	0.9494	0.9599
	Ours	**0.7644**	**0.9843**	**0.9651**	0.9689
STARE (trained on DRIVE)	Marin et al.	—	—	0.9528	—
	Fraz et al.	0.7010	0.9770	0.9495	0.9660
	Li et al.	0.7027	0.9828	0.9545	0.9671
	Yan et al.	0.7211	0.9840	0.9569	**0.9708**
	Ours	**0.7713**	**0.9850**	**0.9684**	0.9694

### 5.6 Segmentation Results

We analyze the segmentation results of our method on the thin vessels and compare the results with those obtained by Yan et al. (2019). In order to make it easy for comparison, as with their method, we separate the thick vessels and thin vessels with a boundary of three pixels, and then we calculate Se, Sp, and Acc of the thin vessel segmentation results. From Table 4, we can see that our Se is lower than the result of the study by Yan et al. (2019), but our Sp and Acc are significantly higher than them, which shows that we have improved the segmentation of thin vessels.

**TABLE 4:** Comparison of thin vessels segmentation.

Method	Se	Sp	Acc
Yan et al. (2019)	**0.8170**	0.8115	0.8127
Our proposed	0.7677	**0.9779**	**0.9602**

**FIGURE 12:**
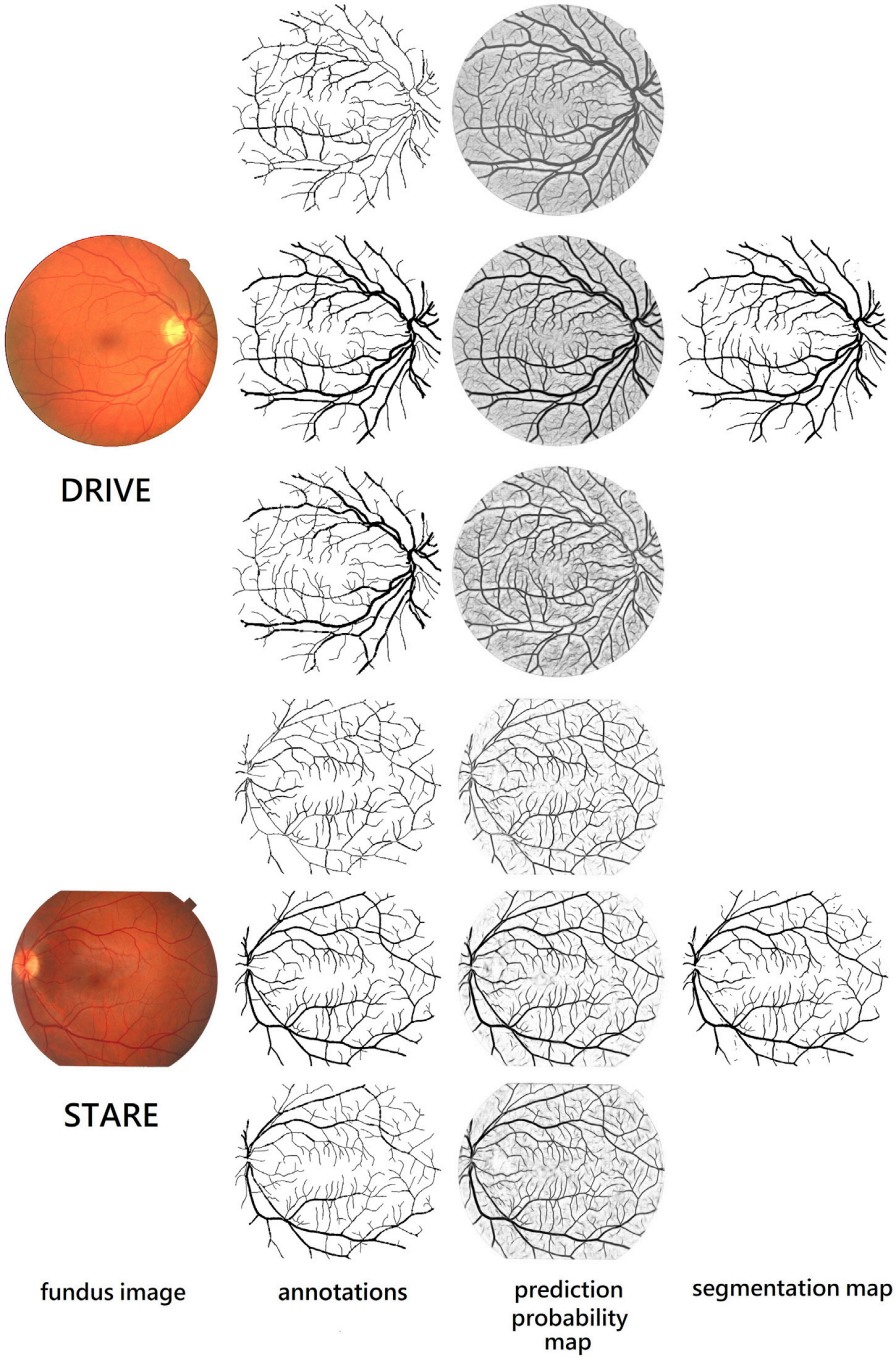
Performance on retina vessel segmentation. From the top to bottom: images from DRIVE and STARE. From the left to right: fundus retina images, ground truths of thin, original, and thick vessels, and prediction probability maps of thin, original, and thick vessels, and binary segmentation map.

We also compare different retina vessel segmentation methods on three datasets, as shown in Table 5. The performances of our proposed method for segmentation retina images are shown in Figure 12. For DRIVE, we achieve 0.9697, 0.8140, 0.9847, 0.8247, and 0.9782 on Acc, Se, Sp, F1-score, and AUC, respectively. Compared to the current excellent experimental results, we surpass them on Acc, Se, and Sp. But for AUC, we differ from the best result, 0.9830 of Wu et al. (2020), by 0.0048. And for the F1-score, our result is lower than that obtained using DEU-net (Wang et al., 2019) by 0.0023, which achieves 0.8270. For STARE, as mentioned above, we use FOV masks generated by Marin et al. (2011). Our framework obtains 0.9737, 0.8251, 0.9859, 0.8239, and 0.9821 on Acc, Se, Sp, F1-score, and AUC, respectively. Compared with many existing methods, our method surpasses current state-of-the-art methods on F1-score and Acc. But for Se, Sp and AUC, we are slightly lower. Among them, AUC is 0.0058 lower than the current best result (Li et al., 2016). For the IOSTAR dataset, we get 0.7998, 0.9847, 0.8059, 0.9702, and 0.9788 on Se, Sp, F1-score, Acc, and AUC, respectively. Compared to the current outstanding experimental results, we surpass them on Sp and AUC. Especially for AUC, we are higher than $CS^{2}$-net (Mou et al., 2021) by 0.003, which achieves 0.9758.

**TABLE 5:** Comparison of different retina vessel segmentation methods on DRIVE, STARE and IOSTAR datasets.

Dataset	Method	Se	Sp	F1	Acc	AUC
DRIVE	2nd observer	0.7760	0.9724	—	0.9472	—
	Zhang et al. (2010)	0.7120	0.9724	—	0.9382	—
	Marin et al. (2011)	0.7067	0.9801	0.7690	0.9452	0.9588
	Fraz et al. (2012)	0.7406	0.9807	—	0.9480	0.9747
	Roychowdhury et al. (2015)	0.7395	0.9782	—	0.9494	0.9672
	Li et al. (2016)	0.7569	0.9816	—	0.9527	0.9738
	Yan et al. (2018)	0.7653	0.9818	—	0.9542	0.9752
	Yan et al. (2019)	0.7631	0.9820	—	0.9538	0.9750
	Liu et al. (2019)	0.8072	0.9780	0.8225	0.9559	0.9779
	Wang et al. (2019)	0.7940	0.9816	**0.8270**	0.9567	0.9772
	Li et al. (2020)	0.7791	0.9831	0.8218	0.9574	0.9813
	Wu et al. (2020)	0.7996	0.9813	—	0.9582	**0.9830**
	**Ours**	**0.8140**	**0.9847**	0.8247	**0.9697**	0.9782
STARE	2nd observer	0.8952	0.9384	—	0.9349	—
	Zhang et al. (2010)	0.7177	0.9753	—	0.9484	—
	Marin et al. (2011)	0.6944	0.9819	0.7531	0.9526	0.9769
	Fraz et al. (2012)	0.7548	0.9763	—	0.9534	0.9768
	Roychowdhury et al. (2015)	0.7317	0.9842	—	0.9560	0.9673
	Yin et al. (2015)	**0.8541**	0.9419	—	0.9325	-
	Li et al. (2016)	0.7726	0.9844	—	0.9628	**0.9879**
	Yan et al. (2018)	0.7581	0.9846	—	0.9612	0.9801
	Yan et al. (2019)	0.7735	0.9857	—	0.9638	0.9833
	Liu et al. (2019)	0.7771	0.9843	0.8036	0.9623	0.9793
	Li et al. (2020)	0.7715	**0.9886**	0.8146	0.9701	0.9881
	Wu et al. (2020)	0.7963	0.9863	—	0.9672	0.9875
	**Ours**	0.8251	0.9859	**0.8239**	**0.9737**	0.9821
IOSTAR	Ronneberger et al. (2015)	0.8044	0.9793	—	0.9675	0.9464
	Azzopardi et al. (2015)	0.7610	0.9670	—	0.9410	0.9550
	Zhao et al. (2018)	0.7720	0.9670	—	0.9480	0.9600
	Alom et al. (2018)	0.8042	0.9779	—	0.9652	0.9530
	Gu et al. (2019)	0.8110	0.9749	—	0.9572	0.9658
	Fu et al. (2019)	0.8298	0.9832	—	0.9720	0.9504
	Mou et al. (2021)	**0.8341**	0.9831	—	**0.9722**	0.9758
	**Ours**	0.7998	**0.9847**	0.8059	0.9702	**0.9788**

In addition, we also calculate the standard deviation of the F1-score obtained by using our method based on U-net and using only U-net on the DRIVE test set. According to Table 6, our standard deviation is 0.01744, while U-net’s standard deviation is 0.02345. This indicates that the F1-score predicted by our method fluctuates less than using U-net alone. For different fundus images, our model is less affected by differences in image quality; thus, we can obtain more stable segmentation maps, which also shows that our method has high reliability.

**TABLE 6:** Comparison of standard deviations on the F1-score.

Method	F1-mean	F1-SD
Normal U-net	**0.8157**	**0.02345**
Ours based on U-net	0.8247	0.01744

## 6 Conclusion

This study proposes a novel deep learning method to train original, thick, and thin vessels. At the same time, we design algorithms for extracting vessel skeleton and separating thick or thin blood vessels. Importantly, we make a novel fusion mechanism that can fuse prediction probability maps from three different types of vessels to obtain final binary segmentation map. Experimental results indicate that our proposed method has outperformed many current outstanding retina vessel segmentation methods on DRIVE, STARE, and IOSTAR datasets. The effectiveness and robustness with different image conditions can make this blood vessel segmentation proposal suitable for retinal image computer analyses such as automated screening for early diabetic retinopathy detection.

## Data Availability

The original contributions presented in the study are included in the article/Supplementary Material; further inquiries can be directed to the corresponding author.
